# Effects of fish oil during hemodialysis on nutritional status and quality of life: a randomized double-blinded trial

**DOI:** 10.29219/fnr.v64.4450

**Published:** 2020-08-03

**Authors:** Chi Zhang, Chang Ge, Junsheng Wang, Dong Sun

**Affiliations:** 1 Department of Nephrology, Xuzhou Medical University, Jiangsu, China; 2 Department of Nephrology, The Affiliated Suqian Hospital of Xuzhou Medical University, Jiangsu, China; 3 Department of Otorhinolaryngology-Head and Neck Surgery, The Affiliated Suqian Hospital of Xuzhou Medical University, Jiangsu, China; 4 Department of Internal Medicine and Diagnostics, Xuzhou Medical University, Jiangsu, China

**Keywords:** fish oil, hemodialysis, PEW, supplement, trail

## Abstract

**Background:**

Supplementation of fish oil has been shown to exert beneficial effects in patients undergoing hemodialysis. The aim of this study was to investigate the efficacy of fish oil in improving the quality of life of these patients through a randomized, double-blinded clinical trial.

**Methods:**

Among the 103 patients enrolled in the study, a total of 74 patients were randomized to receive fish oil (intervention group) or placebo (*n*=37 per group). Patients received identical soft-gel capsules, with each capsule containing either 1000 mg fish oil or placebo for 4 months. Personnel responsible for data collection and analyses were blinded to the grouping.

**Results:**

The reduction of protein-energy wasting (PEW) in the intervention group was significantly more prominent compared to the placebo group (*P*=0.023). The intervention group demonstrated significant increase in midarm circumference, arm muscle circumference, and triceps skinfold thickness after fish oil intake. The intervention group also exhibited significant differences from the placebo group in creatinine, uric acid, and serum calcium levels. Significant improvement was seen regarding the physical role and energy/figure in the intervention group.

**Conclusions:**

Our study demonstrated that fish oil intake in patient undergoing hemodialysis can significantly reduce PEW, and improve physical and biochemical parameters and quality of life, which could provide guidance to clinical management of these patients.

## Popular scientific summary

- This study evaluated the efficacy of fish oil in improving the quality of life of the patients undergoing hemodialysis.
- Our findings suggest that fish oil intake in patient undergoing hemodialysis can significantly reduce protein-energy wasting, improve physical and biochemical parameters and quality of life.
- Our results could provide guidance to clinical management of the patients undergoing hemodialysis.

Chronic kidney disease (CKD) is a massive public health issue, having a surge in incidence globally. Kidney disease has been ranked as the 12th most common cause of death, being responsible for 1.1 million deaths globally according to the 2015 Global Burden of Disease Study (1). An increase of 31.7% in overall CKD mortality has been seen over the last 10 years. The end-stage renal disease (ESRD) has become more prevalent in the incoming decades (1). Patients with CKD, particularly those with ESRD and are undergoing maintenance dialysis therapy (MDT), have a higher risk of protein-energy wasting (PEW) as a result of insufficient intake, superimposed catabolism, inflammation, and uremic toxins, which are common states of nutritional and metabolic dysfunctions (2, 3). PEW has a higher prevalence in patients with CKD stages 4–5 (50–75%) and is linked to higher mortality and morbidity risks and worsening of the quality of life (4). These individuals also have a 2–3 times higher risk of death due to cardiovascular diseases (CVD) compared with the general population (5).

Research has shown that CKD patients have dyslipoproteinemia. These abnormalities can be diagnosed based on serum apolipoprotein measurements. Findings by Lee et al. showed that lipid metabolism became abnormal in patients with CKD of stage >2, which could be exemplified by increased plasma total cholesterol, cholesterol C-III, E, and C-III-HP, very-low-density lipoprotein cholesterol, lipoproteins B, and triglycerides (6). Therefore, nutrient supplementation may play an important role in potentially improving the kidney functions of patients undergoing hemodialysis.

Apart from being a good source of proteins, fish oil is also a valuable source of fatty acids. The long chain n-3 polyunsaturated fatty acid (n-3 PUFA), which can be found in fatty fish and fish oil, exerts ameliorating effects by decreasing platelet aggregation; modifying abnormal lipid metabolism; improving blood pressure, endothelium function, and heart rate; and alleviating inflammation and oxidative stress (7, 8). Studies have shown that patients with high-stage CKD have lower n-3 PUFA levels in the blood than the general population, possibly due to lower dietary intake of n-3 PUFA, in addition to the metabolic changes, inflammation, loss of n-3 PUFA, and malabsorption during dialysis (9, 10).

Recently, several studies have revealed that short- or long-term intervention with omega-3 fatty acids may help in reducing the risk of ESRD and proteinuria and increasing the creatinine clearance rate (11, 12). Experiments have shown that the effects of omega-3 fatty acids on proteinuria, plasma phospholipid docosahexaenoic acid (DHA), and eicosapentaenoic acid (EPA) levels are dose dependent (13, 14). The addition of omega-3 fatty acid supplements to the diet has also been shown to lower the risks of CVD associated with CKD (15, 16). The mechanism may involve modulation of triglycerides, low-density lipoprotein cholesterol (LDL) particles, and blood pressure, while increasing high-density lipoprotein cholesterol (HDL), which is a beneficial factor in patients undergoing hemodialysis.

Other studies have, however, failed to find any significant association between omega-3 and reduction in the risk of renal failure (17, 18). With emerging evidences showing the ameliorating effects of fish oil in patients undergoing hemodialysis (19, 20), the current study aimed at investigating the efficacy of fish oil in improving the quality of life during hemodialysis. This study showed that fish oil intake in patients undergoing hemodialysis significantly reduced PEW and improved certain physical and blood parameters and quality of life and thus can provide guidance to clinical management of these patients.

## Materials and methods

### Patients

A total of 103 eligible patients undergoing hemodialysis, who were admitted to the Affiliated Suqian Hospital of Xuzhou Medical University during 2009–2019, were invited to the study. Eligibility criteria included: out-patients of 18–70 years of age, signed written informed consent, any gender. Exclusion criteria included: amputation and hospitalization in the last 3 months, excessive pallor, femoral fistula, dyspnea, orthopedic, and cognitive compromises. Additionally, patients with regular fish oil intake in the last 3 months were excluded. Patients were recommended a diet plan according to the Kidney Disease Outcomes Quality Initiative (KDOQI) and to maintain a healthy physical activity according to the program “Exercise: A Guide for People on Dialysis” (21). This study was approved by the Ethics Committee of the hospital (Number: 20191101). Written informed consent was obtained from all the patients.

In the intervention group, fish oil (Weihai Ziguang Biological Technology Development Limited Company, Weihai, China) was given daily as three white soft-gel capsules for 4 months. Each capsule contained 1000 mg fish oil (101 mg DHA and 78 mg EPA). Three capsules of placebo were given to the placebo group with meals for 4 months. All participants were provided identical white capsules with a numeric code. All participants were asked to maintain their regular medications and follow their regular diet and level of physical activity during the study period.

### Measurement of anthropometric parameters

Anthropometric parameters, such as weight, height, and triceps skinfold thickness, were measured by a nutritionist with at least 3 years of experience in anthropometric measurement using standard procedures.

### Laboratory parameters

Blood samples from patients were collected before the hemodialysis to measure hemoglobin, white blood cells (WBCs), serum ferritin, total lymphocyte count (TLC), creatinine, uric acid, and calcium using standard lab testing procedures.

### Diagnosis of PEW

PEW was diagnosed when the patients demonstrated three signs: serum albumin of <3.8 g/dL, body-mass index (BMI) of <23 kg/m^2^, and AMA reduction (reduction of >10% in relative to the 50th percentile of the reference).

### Assessment of quality of life

Assessment of quality of life was performed before and after interventions (fish oil or placebo treatment for 4 months). The KDQOL Short Form (KDQOL-SF) contained questions precoded numerically transformable to values of 0–100, with higher score meaning better quality of life. Scores equal to or lower than the mean value were considered indicators of low quality of life.

### Statistical analysis

Student’s *t*-test or Mann–Whitney test was performed for pre- and post-intervention comparisons. A *P*-value of lower than 0.05 was considered statistically significant. Chi-square test or Fisher’s exact test was used for assessing the distribution of observations or phenomena between groups. R software was used for data analysis.

## Results

### Recruitment, randomization, and baseline characteristics

**Fig. 1:**
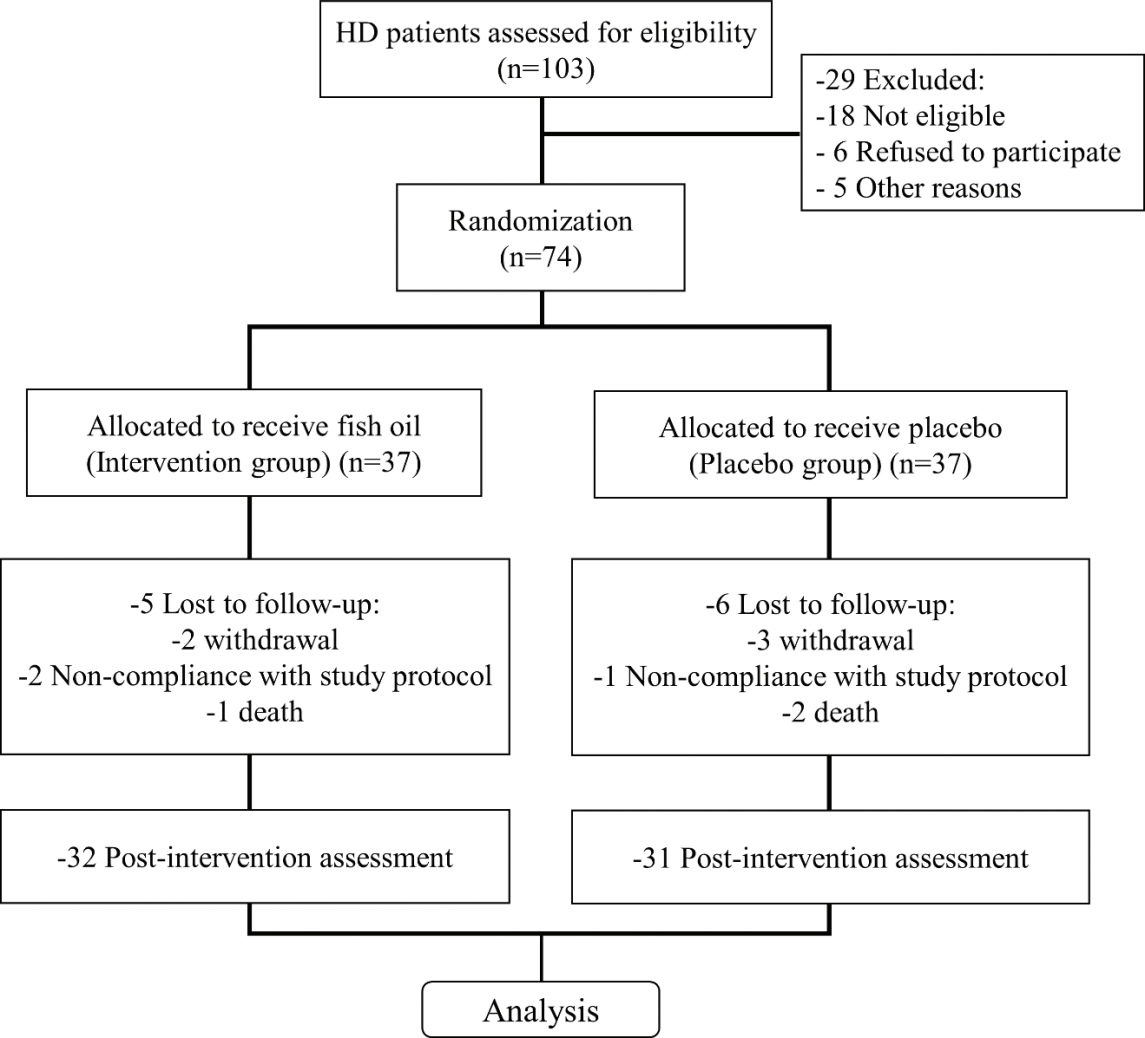
Research framework of this study.

A total of 103 patients undergoing hemodialysis, admitted to the Suqian People’s Hospital during 2009–2019, were assessed for eligibility. Among them, 29 were excluded, 18 were not eligible, 6 refused to participate, and 5 were not included in the study for other reasons. The remaining 74 patients were randomized into two groups, which received either fish oil (intervention group, *n*=37) or placebo (*n*=37). Sequence generation using STATA software was performed for randomization using gender as stratification. After 4 months’ treatment, 32 patients in the intervention group and 31 patients in the placebo group underwent post-intervention analysis (Fig. 1). Subjects who dropped out of the study were those who failed to follow up (5 in the intervention group and 6 in the placebo group), withdrew (2 in the intervention group and 3 in the placebo group), did not comply with the study group (2 in the intervention group and 1 in the placebo group), and died (1 in the intervention group and 2 in the placebo group).

The demographic, biochemical, and clinical characteristics of patients between the two groups are shown in Table 1. The two groups did not significantly differ in terms of demographics, such as age, and their durations and frequency of hemodialysis were similar. The study population was majorly composed of men (59.38% in intervention group and 64.52% in placebo group) and older adults in the age of 40–60 (50.5 ± 12.8 in intervention group and 47.8 ± 15.4 in placebo group). Most patients went through dialysis twice per week (81.25% in the intervention group and 74.19% in the placebo group), while the rest went through dialysis 3 times per week. Social economic statuses, such as education, marriage, and employment, were also similar between the two groups. No statistical significance was found in BMI, and the proportion of patients with chronic diseases, including hypertension, diabetes mellitus, and glomerulopathy, was comparable between the two groups.

**Table 1:** Demographic, clinical, and biochemical characteristics of the patients analyzed

Variable	Study group		*P*
	Intervention group (*n*=32)	Placebo group (*n*=31)	
Age (years)	50.5 ± 12.8	47.8 ± 15.4	0.460
Male gender, *n* (%)	19 (59.38%)	20 (64.52%)	0.797
Body mass index	21.6 ± 4.4	23.1 ± 5.9	0.264
Duration on dialysis (years)	4.4 ± 3.2	3.9 ± 2.7	0.517
Protein-energy wasting	17 (53.13%)	19 (61.29%)	0.613
Etiology, *n* (%)			
Unknown	14 (43.75%)	11 (35.48%)	0.609
Hypertension	6 (18.75%)	9 (29.03%)	0.387
Diabetes mellitus	4 (12.50%)	2 (6.45%)	0.672
Glomerulopathy	3 (9.38%)	5 (16.13%)	0.474
Other	5 (15.62%)	4 (12.91%)	1.000
Frequency of dialysis, *n* (%)			
2 times per week	26 (81.25%)	23 (74.19%)	0.556
3 times per week	6 (18.75%)	8 (25.81%)	0.556
Comorbidities, *n* (%)			
Diabetes	5 (15.62%)	2 (6.45%)	0.426
Hypertension	23 (71.88%)	25 (80.65%)	0.556
Employment status, *n* (%)			
Unemployed	23 (71.88%)	18 (58.06%)	0.297
Employed	9 (28.12%)	13 (41.94%)	0.297
Marital status, *n* (%)			
Married	20 (62.5%)	17 (54.84%)	0.613
Widowed	3 (9.375%)	2 (6.45%)	1.000
Divorced	6 (18.75%)	9 (29.03%)	0.387
Not married	3 (9.375%)	3 (9.68%)	1.000
Educational level, *n* (%)			
<High school	13 (40.63%)	16 (51.61%)	0.453
High school graduate	12 (37.5%)	10 (32.26%)	0.793
College graduate	7 (21.87%)	5 (16.13%)	0.750

Values were expressed as *n* (percentage, %) or mean ± SD. *P*-values for each group were derived from either unpaired *t*-test or Mann–Whitney test as appropriate. Chi-square test or Fisher’s exact test was used for assessing distribution of observations or phenomena between different groups.

### Fish oil treatment alleviates PEW in patients undergoing hemodialysis

As PEW is a common syndrome in patients undergoing chronic hemodialysis, we first analyzed the effects of fish oil intake on PEW. Table 2 shows the prevalence of PEW at baseline and post-intervention in both groups, as a parameter to evaluate the ameliorating effects of fish oil intake. At baseline, the PEW prevalence was 53.13% in the intervention group and 61.29% in the placebo group. After invention, patients with fish oil intake had a dramatically decreased prevalence of PEW (37.5%) compared to those who had placebo (67.74%). The difference comparing post-intervention prevalence in the two groups was significant (*P*=0.023). The changes in both intervention group and placebo group were not significant.

**Table 2:** Protein-energy wasting before and after the intervention in the intervention and placebo groups

Variables		Study group		*P*
		Intervention group (*n*=32)	Placebo group (*n*=31)	
Protein-energy wasting	Baseline	17 (53.13%)	19 (61.29%)	0.613
	Post-intervention	12 (37.5%)	21 (67.74%)	**0.023**
	*P*	0.315	0.603	

### Effects of fish oil intake on body composition and biochemical parameters

The changes in body composition, bioelectric impedance analysis, and muscle strength are shown in Table 3. Among the weight, BMI, arm muscle circumference (AMC), midarm circumference (MAC), arm muscle area (AMA), triceps skinfold thickness, fat mass as percentage of body weight (FM%), handgrip strength, resistance at 50 kHz (R), reactance at 50 kHz (Xc) and phase angle, MAC, AMC, and triceps skinfold thickness demonstrated significant changes after intervention in patients with fish oil intake, the post-intervention values of which, along with handgrip strength, also showed significant difference compared to those of the placebo group.

**Table 3:** Changes in body composition measured by anthropometrics, muscle strength (dynamometry), and bioelectric impedance analysis

Variables		Study group		*P*
		Intervention group (*n*=32)	Placebo group (*n*=31)	
Weight (kg)	Baseline	46.2 ± 6.8	48.5 ± 7.3	0.209
	Post-intervention	49.1 ± 7.3	46.9 ± 8.2	0.272
	*P*	0.223	0.518	
Body mass index (kg/m^2^)	Baseline	21.6 ± 4.4	23.1 ± 3.1	0.129
	Post-intervention	22.2 ± 3.9	22.7 ± 2.0	0.521
	*P*	0.178	0.592	
Midarm circumference (cm)	Baseline	23.8 ± 2.5	23.4 ± 1.9	0.487
	Post-intervention	27.6 ± 3.2	22.9 ± 2.3	**<0.0001**
	*P*	**0.0001**	0.454	
Arm muscle circumference (mm)	Baseline	202.8 ± 17.3	204.7 ± 14.7	0.645
	Post-intervention	221.4 ± 16.7	206.5 ± 10.5	**0.0001**
	*P*	**<0.0001**	0.634	
Arm muscle area (cm^2^)	Baseline	31.6 ± 2.3	31.1 ± 2.2	0.391
	Post-intervention	31.3 ± 1.4	30.9 ± 2.4	0.437
	*P*	0.449	0.784	
Triceps skinfold thickness (mm)	Baseline	8.1 ± 1.3	7.8 ± 1.6	0.425
	Post-intervention	11.4 ± 2.8	7.4 ± 1.7	**<0.0001**
	*P*	**<0.0001**	0.124	
Fat mass as percentage of body weight from anthropometry	Baseline	17.9 ± 2.8	17.9 ± 3.0	0.996
	Post-intervention	18.2 ± 2.9	18.7 ± 3.5	0.549
	*P*	0.751	0.462	
Handgrip strength (kg)	Baseline	16.2 ± 2.6	15.7 ± 3.5	0.530
	Post-intervention	16.9 ± 2.8	14.1 ± 3.2	**0.005**
	*P*	0.114	0.154	
Resistance at 50 kHz (ohm)	Baseline	584.3 ± 36.1	576.8 ± 31.8	0.392
	Post-intervention	598.5 ± 29.8	592.6 ± 32.0	0.457
	*P*	0.167	0.097	
Reactance at 50 kHz (ohm)	Baseline	63.2 ± 5.6	63.1 ± 6.8	0.951
	Post-intervention	64.8 ± 6.2	65.9 ± 6.7	0.508
	*P*	0.394	0.220	
Phase angle (°)	Baseline	5.8 ± 1.2	5.9 ± 1.3	0.749
	Post-intervention	6.1 ± 1.1	6.1 ± 1.3	0.991
	*P*	0.171	0.343	

Values were expressed as mean ± SD. *P*-values derived from paired *t*-test or Wilcoxon signed rank test as appropriate between baseline and post-intervention. *P*-values derived from unpaired *t*-test or Mann–Whitney test as appropriate between intervention group and placebo group.

Among the biochemical parameters measured shown in Table 4, which included the level of hemoglobin, WBC count, serum ferritin, total lymphocytes count, creatinine, uric acid, Hs.CRP, calcium, phosphorus, ALP, and OpG, creatinine demonstrated significant decrease in the intervention group, while calcium levels demonstrated significant increase. While no prominent changes in uric acid level were observed in the intervention group, a significant increase of uric acid level was observed in the placebo group. The post-intervention levels of creatinine and uric acid were significantly different between the intervention group and the placebo group. No statistically significant differences in other levels were documented.

**Table 4:** Changes in laboratory parameters in the intervention and placebo groups

Variables		Study group		*P*
		Intervention group (*n*=32)	Placebo group (*n*=31)	
Hemoglobin (g/dL)	Baseline	9.6 ± 2.5	9.3 ± 2.8	0.664
	Post-intervention	9.9 ± 1.9	10.1 ± 3.0	0.762
	*P*	0.669	0.072	
White blood cells (×10^9^/L)	Baseline	5.3 ± 1.7	5.4 ± 1.8	0.830
	Post-intervention	5.8 ± 1.8	5.7 ± 1.6	0.819
	*P*	0.391	0.587	
Serum ferritin (ng/mL)	Baseline	564 ± 34	561 ± 32	0.722
	Post-intervention	582 ± 49	574 ± 45	0.510
	*P*	0.142	0.186	
Total lymphocytes count (cells/mm^3^)	Baseline	953 ± 73	984 ± 53	0.064
	Post-intervention	974 ± 78	965 ± 55	0.601
	*P*	0.381	0.180	
Creatinine (mg/dL)	Baseline	16.2 ± 4.2	16.9 ± 3.7	0.492
	Post-intervention	11.6 ± 3.5	17.8 ± 4.5	**<0.0001**
	*P*	**<0.0001**	0.140	
Uric acid (mg/dL)	Baseline	7.6 ± 1.6	7.3 ± 1.3	0.429
	Post-intervention	7.3 ± 1.8	8.8 ± 1.5	**0.0008**
	*P*	0.257	**<0.0001**	
Calcium (mg/dL)	Baseline	8.9 ± 1.5	9.1 ± 1.3	0.580
	Post-intervention	10.2 ± 1.8	9.8 ± 1.9	0.402
	*P*	**0.011**	0.063	

Values were expressed as mean ± SD as appropriate. *P*-values derived from paired *t*-test or Wilcoxon signed rank test as appropriate between baseline and post-intervention. *P*-values derived from unpaired *t*-test or Mann–Whitney test as appropriate between intervention group and placebo group.

### Quality of life assessment

The assessment based on the KDQLQ-SF questionnaire shown in Table 5 suggests that patients in the intervention group generally showed higher scores in majority of the items in the questionnaire, in comparison with a decrease of scores in the placebo groups. Significant improvement was seen in energy or fatigue in the intervention group, and the post-intervention value was significantly different from the placebo group.

**Table 5:** Assessment of quality of life before and after the intervention by KDQOL-short form questionnaire

Variables		Study group		*P*
		Intervention group (*n*=32)	Placebo group (*n*=31)	
Physical function	Baseline	58 ± 11	61 ± 12	0.318
	Post-intervention	64 ± 13	59 ± 10	0.097
	*P*	0.123	0.342	
Physical role	Baseline	35 ± 8	38 ± 6	0.102
	Post-intervention	69 ± 14	36 ± 8	**<0.0001**
	*P*	**<0.0001**	0.094	
Emotional role	Baseline	64 ± 17	69 ± 18	0.273
	Post-intervention	67 ± 13	65 ± 15	0.580
	*P*	0.223	0.184	
Energy/fatigue	Baseline	59 ± 14	54 ± 13	0.153
	Post-intervention	71 ± 14	50 ± 11	**<0.0001**
	*P*	**0.010**	0.296	

Values were expressed as mean ± SD. *P*-values derived from paired *t*-test or Wilcoxon signed rank test as appropriate between baseline and post-intervention. *P*-values derived from unpaired *t*-test or Mann–Whitney test as appropriate between intervention group and placebo group.

## Discussions

Previous studies on fish oil supplementation among patients undergoing chronic hemodialysis therapy suggest that nutrient supplementation may be of great benefit for health and quality of life of patients. This study is a randomized, double-blinded clinical trial set to investigate the efficacy of fish oil in improving the quality of life of these patients. Of the 103 patients recruited, 32 in the intervention group and 31 in the placebo completed the post-intervention analysis after 4 months of intervention. The two groups were comparable in terms of gender composition, duration of hemodialysis, and age. For both arms of the study, the majority of the patients also had dialysis twice per week. The similarity of the two arms in these demographics allows for the comparison of the two groups in further analysis without any adjustments, as differences in these demographics would affect the analysis (22).

PEW has previously been identified as a common syndrome in patients undergoing chronic hemodialysis (23). Analysis of the effects of fish oil intake on PEW showed a significant difference between the intervention group and the placebo group. This finding is in agreement with other previous studies, where the use of fish oil by patients on dialysis ameliorates protein wasting (24). The fish oils are rich in omega 3 fatty acids that participate in a number of metabolic events including anti-inflammatory effects (25). DHA and EPA and also exert muscle anabolic actions regardless of their anti-inflammatory actions, which suggests that this could be a potential therapeutic tool for alleviating PEW (26, 27).

PEW of kidney diseases is characterized by loss of skeletal muscle concomitant with decreased visceral protein concentrations (28). We also observed a difference in the body composition evidenced by muscle strength (dynamometry), anthropometrics, and bioelectric impedance analysis when we compared at baseline and post-intervention to the placebo group. The potential mechanisms by which fish oil modulates muscle protein turnover have not been clarified. Some studies have indicated that this could be through a pronounced effect on protein synthesis (26, 29), whereas other studies documented a decrease in protein breakdown (27) or negligible effect on protein turnover (30).

Recent studies have strived to extend the use of Modification of Diet in Renal Disease Equations (MDRDEs) to ESRD. A strong correlation was shown between MDRDE and creatinine clearance (31). Nonetheless, Virga et al. uncovered a correlation between creatinine clearance and a number of glomerular filtration rate-estimating equations during dialysis (32). The findings in this study showed that there was a significant decrease in creatinine levels for individuals in the intervention group, indicating renal recovery in this group. These findings are in agreement with that of Lauretani et al. who demonstrated that older adults with low total plasma polyunsaturated fatty acid levels had a more pronounced decrease in creatinine clearance, suggesting that a higher dietary intake of polyunsaturated fatty acids confers protection against progression to CKD (33).

Quality of life was another parameter which we assessed in our patients in order to see whether fish oil had beneficial effects. Findings from our study showed that patients in the intervention group generally showed higher scores in the majority of the items in the questionnaire in comparison with a decrease of scores in the placebo groups, thereby confirming that fish oil can improve the quality of life of ESRD patients on chronic hemodialysis. This finding is in agreement with that of Moeinzadeh et al. who demonstrated an improved quality of life in ESRD patients on chronic hemodialysis who had omega-3 supplementation in their daily dietary program (34).

The findings of this study have demonstrated that intake of fish oil in patient undergoing hemodialysis can significantly reduce PEW, and improve certain physical and blood parameters and quality of life, and this can provide a guideline for the clinical management of these patients. The limitation of the study is that the mechanism of fish oil in improving nutritional status of patient undergoing hemodialysis is not investigated, and further studies are needed to study the mechanism of action. In addition, here the intervention group received fish oil for 4 months, and whether shorter or longer duration of fish oil intake would strengthen the improvement for patients undergoing hemodialysis is unknown, which is worth investigating in future studies.

## Conclusions

Our clinical trial has demonstrated that fish oil intake in patient undergoing hemodialysis can significantly reduce PEW, and improve certain physical and blood parameters and quality of life, which could provide guidance to clinical management of these patients.

## Conflict of interest and funding

The authors declare no conflicts of interest. They have not received any funding or benefits from industry or elsewhere to conduct this study.

## Authors’ contributions

Chi Zhang, Chang Ge, and Junsheng Wang contributed to the data collection and analysis. Dong Sun contributed to the project development and manuscript writing.
